# Parent–child cooking meal together may relate to parental concerns about the diets of their toddlers and preschoolers: a cross-sectional analysis in Japan

**DOI:** 10.1186/s12937-019-0480-0

**Published:** 2019-11-18

**Authors:** Midori Ishikawa, Kumi Eto, Miki Miyoshi, Tetsuji Yokoyama, Mayu Haraikawa, Nobuo Yoshiike

**Affiliations:** 1 Department of Health Promotion, National Institute of Public Health, 2-3-6 Minami, Wako, Saitama, 351-0197 Japan; 2 Faculty of Nutrition, Kagawa Nutrition University, 3-9-21 Chiyoda, Sakado, Saitama, 350-0288 Japan; 3 Department of Nutrition, Faculty of Health Sciences, Aomori University of Health and Welfare, 58-1 Mase, Hamadate, Aomori, 030-8505 Japan; 4 Department of Child Studies, Faculty of Child Studies, Seitoku University, 550 Iwase, Matsudo, Chiba, 271-8555 Japan

**Keywords:** Cooking together, Child, Parent, Picky eating, Playing with food/utensils while eating, Eating too much

## Abstract

**Background:**

Parents often have concerns about the food habits of their young children. Cooking is a frequent behavior related to dietary activities at home. We hypothesized that “a parent cooking meals together with young children might alleviate dietary concerns.” The aim of this study was to identify the relationship between parental cooking practices (e.g., cooking meals together with the child) and diet-related concerns.

**Methods:**

Data were extracted from the “National nutrition survey on preschool children” conducted among nation-wide households with toddlers and preschoolers in 2015 by the Ministry of Health, Labour and Welfare of Japan. Parents were classified into two groups comprising those who cooked meals together with their children and those who did not. The following variables were compared: taking too much time to eat (slow eaters), “picky” eating (eating only certain foods), inconsistent food intake (eating too much or too little), playing with food/utensils while eating, preferring sweetened beverages and snacks over meals, eating too fast to chew well, not swallowing food, disinterested in eating, and spitting out food. The associations between parent–child cooking meals together and the concerns pertaining to the child’s dietary habits and food intake were analyzed and compared between the two groups.

**Results:**

The concerns of “picky eating” and “playing with food/utensils while eating” were lower, while “eating too much” was higher in the parent-cooking together group. The intake frequency of fish, soybeans/soy products, vegetables, and milk among children were higher in the “cooking together” group than among those in the “not cooking together” group. Children in the “cooking together” group consumed a significantly greater variety of foods than those in the “not cooking together” group.

**Conclusions:**

Cooking a meal together with a child may be related to the parent’s lower concerns about the dietary habits of the child, including “picky eating” and “playing with food/utensils while eating,” but may also be related to the higher concerns of “eating too much.”

**Electronic supplementary material:**

The online version of this article (10.1186/s12937-019-0480-0) contains supplementary material, which is available to authorized users.

## Background

Dietary intake and eating habits developed in early childhood affect the development of school-aged children [1]. Furthermore, eating behaviors affect health and nutritional status in adulthood, and the onset of lifestyle-related diseases [2–4].

According to the “National nutrition survey on preschool children” conducted in Japan [5], about 80% of parents expressed frequent concerns about the dietary habits of their children, such as “taking too much time to eat,” “picky eating (eating only certain foods),” “inconsistent food intake (eating too much or too little, depending on the day,”) “playing with food/utensils while eating,” “preferring sweetened beverages and snacks over meals,” and “eating too fast to chew well.”

There have been several reports on how parents can alleviate dietary-related concerns in school-aged children. Some claim that cooking a meal together with others in school or preschool is an effective way to improve “picky eating,” “eating too fast,” and “preferring sweetened beverages and snacks over meals” [6–8].

Another study reported that the dislike of vegetables and picky eating had improved with classes and practical sessions, including lessons in the school curriculum on cooking meals, as well as food and nutrition (*shokuiku* in Japanese) [9]. Even in early childhood, some nursery schools and kindergartens have incorporated parent–child cooking classes in *shokuiku* programs, and reported that this strategy can effectively improve a child’s dislike of vegetables [9, 10].

However, the formation of dietary behaviors in the preschool period might be related to the dietary habits of the parents [11–15]. Cooking is a repeated behavior related to dietary practices at home and some parents may cook a meal together with a young child. Nevertheless, relatively few studies have investigated the influence of parent–child cooking activities at home on the dietary behaviors of children and whether such activities may relate to parental concerns regarding the dietary habits of young children [6].

We hypothesized that “a parent cooking meals together with a young child” may relate to parental concerns about the dietary habits of their children, such as “taking too much time to eat,” “eating only certain foods,” “eating inconsistent amounts of food,” “playing with food/utensils while eating,” “preferring sweetened beverages and snacks over meals,” and “eating too fast to chew well” as well as “inconsistent food intake.”

## Methods

### Study population and procedure

Data were retrieved from the “National nutrition survey on preschool children,” which was conducted in September 2015 by the Ministry of Health, Labour, and Welfare, Japan (MHLW) [5]. Children aged ≤6 years as of May 31, 2015 were randomly selected from households in Japan among 1106 districts for the “Comprehensive Survey of Living Conditions.” The three districts affected by heavy rain in September 2015 were excluded from the survey sampling. First, the MHLW explained the survey method to the prefectures. Subsequently, the prefectural public health center employed investigators to visit the households selected for this survey. The investigators asked the child’s mother (or the caregiver involved in providing meals to the child) to complete a questionnaire, which was collected at a later date. In total, 2992 households with 3936 children aged ≤6 years participated in the survey. The questionnaires of 65 children were excluded because information on age was not available. Finally, 3871 questionnaires were collected for analysis [5]. A database was prepared by the Maternal and Child Health Division, Department of Equal Employment and Children’s Family, MHLW.

The “National nutrition survey on preschool children” has two types of questionnaires: one for infants aged < 2 years and the other for children aged 2–6 years. Data obtained from the latter questionnaire were used in this study. In total, 2237 persons responded to all items consistent with the purpose of this study (Additional file 1).

### Measurement

The objective variable of this study, parent–child cooking meals together, was posed as a question to the participants: “Do you (parent) try to cook a meal together with your child?” The parent answered with a “yes” or “no.” Explanatory variables related to parents included relationships with the children, age of mother, current employment status of mother, structure of family members living together, subjective economic status, and leisure time (affluent, somewhat, neither, not so much, not at all) etc.

Parental concerns about the daily dietary habits of their children consisted of 11 items: “taking too much time to eat (slow eaters),” “picky” eating (eating only certain foods),” “the amount of inconsistent food intake (too much or too little, depending on the day),” “playing with food/utensils while eating,” “preferring sweetened beverages and snacks over meals,” “too little food intake,” “eating too fast to chew well,” “not swallowing food,” “disinterested in eating, “eating too much,” and “spitting out food.” For all questions, the parent answered with a “yes” or “no.”

As for children, data of age, height, weight, nutritional status (degree of obesity), food allergies, tooth decay, and time spent on TV, video, and games were obtained. Furthermore, the food diversity score (FDS) was used to assess the nutritional quality of the whole diet [16]. The typical eating patterns of eight food groups (grains, fish, meat, eggs, soybeans/soy products, vegetables, fruit, and milk) were evaluated. Moreover, the intake of processed foods was also examined, in which four items (sweetened beverages, confectioneries, instant noodles, and fast food) were investigated by inquiring how often the participants consumed foods in each group (≥2 times per day, once a day, 4–6 days per week, 1–3 days per week, and less than once a week or rarely).

### Nutritional status of children

The nutritional status of children was determined based on body weight and height. The degree of obesity (%) was calculated using the following formula: self-reported body weight (g) − standard body weight (g) for height/standard body weight (g) for height × 100. The judgment criteria for the degree of obesity were “obese” (≥30%), “overweight” (20–30%), “tendency to be overweight” (15–20%), “standard” (− 15 to + 15%), “tendency to be underweight”: (less than − 15% to less than − 20%), and “underweight” (less than − 20%). The standard body weight was calculated using the formula of standard body weight for height in Japanese children [17, 18]. The formula does not consider age because the standard body weight for height curves were almost identical for children aged 1–6 years [19].

### Statistical analysis

By classifying the parents into two groups, parents who cooked meals together with their children (“cooking together”) and those who did not (“not cooking together”), the parents’ sex, age of mother, and socioeconomic status as well as the sex, nutritional status, food allergies, tooth decay, and time spent on TV, video, and games of children were compared.

Next, the 11 concerns of parents on the dietary habits of their children were compared between the two groups. Multivariate analysis was performed for each of the 11 concerns using a logistic regression model, adjusted for the relationship with the child (mother or father), child’s sex, employment status of mother (yes or no), and family members in the household (other children, grandparents, and others) (Model 1).

Additional multivariate analysis was performed for each of the 11 concerns using a logistic regression model, adjusted for the relationship with the child (mother or father), child’s sex, employment status of mother (yes or no), family living together (other children, grandparents, and others), subjective economic status (affluent, somewhat, neither, not so much, or not able to afford at all), leisure time (affluent, somewhat, neither, not so much, not at all), and place where the child spends time during the day (nursery school, kindergarten, center for early childhood education and care, with grandparents, with relatives, staying at home) (Model 2).

Finally, the frequency of food intake between the two groups was compared. The FDS was the total number of eight food groups (grains, fish, meat, eggs, soybeans/soy products, vegetables, fruits, and milk) being consumed at least once a day, and the processed food score was the total number of four food items (sweetened beverages, confectioneries, instant noodles, and fast food) being consumed at least once a day [16]. The FDS and the processed food score were compared between the two groups [16]. The FDS was 1 point if once or more a day or 0 points if less than that. There were eight types of food, thus the maximum score was eight points. Similarly, the processed food score was calculated as a score of 1 point if at least once a day or 0 points if less than that. There were four types of food, so the maximum score was four points. All statistical analyses were performed using SAS software, version 9.2 (SAS Institute, Inc., Cary, NC, USA). A probability (*p*) value of < 0.05 was considered statistically significant.

## Results

Table 1 compares the characteristics of mother’s age, employment status, family members living together, subjective economic status or leisure time, and the place where and how the child spends during the day between the two groups. The “cooking together” group had more leisure time than the “not cooking together” group (*p* = 0.0002). However, there were no significant differences in the other variables between the two groups.

**Table 1:** Demographics of parent and household by the parent-child cooking together group

	not cooking together		cooking together		
	n	%	n	%	
	2004	89.6	233	10.4	p
Relationship for the child					
mother	1957	97.7	231	99.1	0.231
father	47	2.4	2	0.9	
	mean	SD	mean	SD	p
Age of mother (years old)†					
mean, SD	36.0	5.1	35.4	5.5	0.101
	n	%	n	%	p
Employment status of mother					
currently work					
yes	1135	56.6	132	56.7	0.996
no	869	43.4	101	43.4	
Family living together					
mother or father and one child	75	3.7	14	6.0	0.365
mother or father and grandparent and one child	64	3.2	7	3.0	
mother and father and one child	314	15.7	44	18.9	
mother and father and children	1209	60.3	128	54.9	
mother and father and grandparent and children	339	16.9	40	17.2	
others (living together with non-family adults)	3	0.2	0	0.0	
Subjective economic status					
affluent	155	7.7	26	11.2	0.357
somewhat	423	21.1	42	18.0	
neither	668	33.3	80	34.3	
not so much	580	28.9	63	27.0	
no afford not at all	178	8.9	22	9.4	
Leisure time					
affluent	144	7.2	36	15.5	0.0002
somewhat	452	22.6	59	25.3	
neither	459	22.9	42	18.0	
not so much	746	37.2	74	31.8	
no afford not at all	203	10.1	22	9.4	
Caregiver of the child during the day					
Nursery school					
yes	827	41.3	98	42.1	0.816
no	1177	58.7	135	57.9	
Kindergarten					
yes	747	37.3	91	39.1	0.595
no	1257	62.7	142	90.9	
Centers for Early Childhood Education and Care					
yes	126	6.3	16	6.9	0.731
no	1878	93.7	217	93.1	
Grandparents and relatives					
yes	105	5.2	8	3.4	0.234
no	1899	94.8	205	96.6	
None of the above					
yes	248	12.4	29	12.5	0.975
no	1756	87.6	204	87.6	
Dietary lifestyle in mother/father					
Eating breakfast					
everyday	1878	93.7	218	93.6	0.423
4-5 days per week	100	5.0	15	6.4	
2-3 days per week	5	0.3	0	0.0	
1 day or less per week	20	1.0	0	0.0	
I do not eat at all	1	0.1	0	0.0	

p: χ^2^ test

†: t test

Table 2 compares the characteristics of the child’s age, sex, nutritional status, food allergies, tooth decay, and the time spent on TV, video, or games between the two groups. In the “cooking together” group, the proportions of children spending less than 2 h per day on TV, video, or games during the weekdays (*p* = 0.025) and weekends (*p* = 0.015) were higher than those in the “not cooking together” group.

**Table 2:** Characteristics of the child’s age, sex, nutritional status, food allergies, tooth decay, and the time spent on TV, video or games by the parent-child cooking together group

	not cooking together		cooking together		
	*n*=2004		*n*=233		
	mean	SD	mean	SD	p
Age †					
years old	4.2	1.1	4.3	1.1	0.310
	n	%	n	%	p
Sex					
male	1062	52.9	96	41.2	0.001
female	944	47.1	137	58.8	
Nutritional status					
+30%≤ (obesity)	10	0.5	4	1.7	0.731
+30 to 20% (overweight)	26	1.3	2	0.9	
+15 to 20% (overweight tendency)	50	2.5	8	3.4	
−15 to+15% (standard)	1870	93.3	208	89.3	
−15 to −20%(underweight tendency)	36	1.8	4	1.7	
≤−20% (underweight)	12	0.6	7	3.0	
Food allergy symptoms					
yes	319	15.9	42	18.0	0.408
no	1685	84.1	191	82.0	
Tooth decay					
yes	384	19.2	36	15.6	0.186
no	1619	80.8	195	84.4	
Time spent on TV, video or games					
weekday					
None	25	1.3	6	2.6	0.025
< 2 hours /day	1512	75.5	188	80.7	
≥ 2 hours /day	467	23.3	39	16.7	
weekend					
None	15	0.8	5	2.2	0.015
< 2 hours /day	1147	57.2	147	63.1	
≥ 2 hours /day	842	42.0	81	34.8	

p: χ^2^ test

†: t test

Table 3 presents the proportions of the parental concerns about the child’s dietary habits (11 items) for both groups. The items are depicted in the order that each was identified as a parental concern. The item with the highest proportion of participants answering “yes” was “taking too much time to eat (slow eaters)” (33.1%), followed by “picky eating (eating only certain foods)” (31.0%), “inconsistent food intake” (25.0%), “playing with food/utensils while eating” (23.8%), “preferring sweetened beverages and snacks over meals” (18.2%), “small food intake” (15.9%), “eating too fast to chew well” (8.9%), “not swallowing food” (6.2%), “disinterested in foods” (5.5%), “eating too much” (4.9%), and “spitting out food” (4.2%).

**Table 3:** Parental concerns about the child’s diet (11 items) by child–parent cooking a meal together group

Parental concerns about the diets of the child			total		not cooking together		cooking together		p
			n	%	n	%	n	%	
			2237	100	2004	89.6	233	10.4	
1	He/she takes too much time for eating (eats slowly).	yes	740	33.1	667	33.3	73	31.3	0.549
		no	1547	69.2	1387	66.7	160	68.7	
2	He/she is a picky eater (eating only certain foods).	yes	693	31.0	635	31.7	58	24.9	0.034
		no	1544	69.0	1369	68.3	175	75.1	
3	The amount of food intake is inconsistent (too much or too little, depending on the day).	yes	559	25.0	503	25.1	56	24.0	0.722
		no	1678	75.0	1501	74.9	177	76.0	
4	He/she plays with food/utensils while eating.	yes	533	23.8	494	24.7	39	16.7	0.007
		no	1704	76.2	1510	75.4	194	83.3	
5	He/she desires sweetened beverage or sweets more than a meal.	yes	408	18.2	369	18.4	39	16.7	0.531
		no	1829	81.8	1635	81.6	194	83.3	
6	The amount of food intake is small.	yes	355	15.9	323	16.1	32	13.7	0.346
		no	1882	84.1	1681	83.9	201	86.3	
7	His/her eats too fast to chew well.	yes	200	8.9	183	9.1	17	7.3	0.353
		no	2037	91.1	1821	90.9	216	92.7	
8	He/she stores the food in his/her mouth.	yes	139	6.2	124	6.2	15	6.4	0.881
		no	2098	93.8	1880	93.8	218	93.6	
9	He/she is not interested in eating.	yes	122	5.5	115	5.7	7	3.0	0.082
		no	2115	94.5	1889	94.3	226	97.0	
10	He/she eats too much.	yes	110	4.9	92	4.6	18	7.7	0.036
		no	2127	95.1	1912	95.4	215	92.3	
11	He/she takes food out of his/her mouth.	yes	95	4.2	86	4.3	9	3.9	0.759
		no	2142	95.8	1918	95.7	224	96.1	

p: χ^2^ test

In the “not cooking together” group, the proportion of parents agreeing to the concern about their children “picky eating (eating only certain foods)” (*p* = 0.034) and “playing with food/utensils eating a meal while eating” (*p* = 0.007) was higher, and “he/she eats too much” (*p* = 0.036) was lower, than in the “cooking together” group.

Table 4 shows the results of the associations between “parental concerns about the diets of the child” and “parent and child cooking together” using step-wise multivariate analysis. In Model 1, the analysis showed that three factors were associated with parent–child cooking together: not picky eating (odds ratio [OR] = 1.39; 95% confidence interval [CI] = 1.02–1.91; *p* = 0.039), not playing with food/utensils while eating (OR = 1.59; 95% CI = 1.11–2.28; *p* = 0.013), and not eating too much (OR = 0.57; 95% CI = 0.34–0.97; *p* = 0.040). In Model 2, the analysis also showed that the three factors were significantly associated with parent–child cooking together were not picky eating (OR = 1.39; 95% CI = 1.01–1.91; *p* = 0.041), not playing with food/utensils while eating (OR = 1.56; 95% CI = 1.08–2.25; *p* = 0.017), and not eating too much (OR = 0.55; 95% CI = 0.32–0.94; *p* = 0.030).

**Table 4:** Associations between “parental concerns about the diets of the child” and “parent and child cooking together”

Parental concerns about the diets of the child (Dependent variable)			Child–parent cooking a meal together (Independent variable)							
			Model 1				Model 2			
			OR	95%CI		p	OR	95%CI		p
1	He/she takes too much time for eating (eats slowly).	yes	1.00				1.00			
		no	1.14	0.85	1.53	0.396	1.11	0.83	1.50	0.478
2	He/she is a picky eater (eating only certain foods).	yes	1.00				1.00			
		no	1.39	1.02	1.91	0.039	1.39	1.01	1.91	0.041
3	The amount of food intake is inconsistent (too much or too little, depending on the day).	yes	1.00				1.00			
		no	1.02	0.74	1.41	0.886	1.01	0.73	1.39	0.972
4	He/she plays with food while eating.	yes	1.00				1.00			
		no	1.59	1.11	2.28	0.013	1.56	1.08	2.25	0.017
5	He/she desires sweetened beverage or sweets more than a meal.	yes	1.00				1.00			
		no	1.13	0.79	1.63	0.499	1.13	0.78	1.64	0.513
6	The amount of food intake is small.	yes	1.00				1.00			
		no	1.22	0.82	1.81	0.328	1.22	0.82	1.82	0.324
7	His/her eats too fast to chew well.	yes	1.00				1.00			
		no	1.21	0.72	2.04	0.479	1.19	0.70	2.01	0.526
8	He/she stores the food in his/her mouth.	yes	1.00				1.00			
		no	0.93	0.53	1.62	0.786	0.91	0.52	1.60	0.735
9	He/she is not interested in eating.	yes	1.00				1.00			
		no	1.86	0.85	4.05	0.120	1.71	0.78	3.75	0.178
10	He/she eats too much.	yes	1.00				1.00			
		no	0.57	0.34	0.97	0.040	0.55	0.32	0.94	0.030
11	He/she takes food out of his/her mouth.	yes	1.00				1.00			
		no	1.07	0.53	2.16	0.855	0.94	0.46	1.92	0.859

Model 1: adjusted relationship for the child, sex of child, employment status of mother, family living together

Model 2: adjusted relationship for the child, sex of child, employment status of mother, family living together, subjective economic status, leisure time, caregiver of the child during the day

*OR* Odds ratio, *CI* Confidence interval

Table 5 shows the food intake frequency of children for both groups. In the “cooking together” group, the intake frequency of fish (*p* = 0.011), soybeans/soy products (*p* = 0.001), vegetables (*p* = 0.003), and milk (*p* = 0.002) was higher than that in the “not cooking together” group. On the other hand, the frequency of eating fast food was lower in the “cooking together” group than in the “not cooking together” group (*p* = 0.014). Furthermore, the FDS was higher in the “cooking together” group than in the “not cooking together” group (*p* = 0.002).

**Table 5:** Frequency of food intake by the parent-child cooking together group

food category	frequency	not cooking together		cooking together		p
		*n*=2004		*n*=233		
		n	%	n	%	
Food group						
grain	≥ 2 times per day	1953	97.5	231	99.1	0.167
	Once a day	35	1.8	1	0.4	
	4–6 days /week	12	0.6	1	0.4	
	1–3 days /week	2	0.1	0	0.0	
	less than once a week	2	0.1	0	0.0	
	have not eaten yet	0	0.0	0	0.0	
fish	≥ 2 times per day	105	5.2	19	8.2	0.011
	Once a day	230	11.5	33	14.2	
	4–6 days /week	466	23.3	56	24.0	
	1–3 days /week	1076	53.7	116	46.8	
	less than once a week	125	6.2	8	3.4	
	have not eaten yet	2	0.1	1	0.4	
meat	≥ 2 times per day	245	12.2	41	17.6	0.140
	Once a day	396	19.8	52	22.3	
	4–6 days /week	905	45.2	83	35.6	
	1–3 days /week	434	21.7	52	22.3	
	less than once a week	23	1.2	3	1.3	
	have not eaten yet	1	0.1	2	0.9	
eggs	≥ 2 times per day	79	3.9	13	5.6	0.279
	Once a day	436	21.8	64	27.5	
	4–6 days /week	708	35.3	76	32.6	
	1–3 days /week	604	30.1	63	27.0	
	less than once a week	144	7.2	13	5.6	
	have not eaten yet	33	1.7	4	1.7	
soybeans and soy products	≥ 2 times per day	136	6.8	24	10.3	0.001
	Once a day	411	20.5	53	22.8	
	4–6 days /week	616	30.7	74	31.8	
	1–3 days /week	701	35.0	65	27.9	
	less than once a week	138	6.9	14	6.0	
	have not eaten yet	2	0.1	3	1.3	
vegetables	≥ 2 times per day	1064	53.1	143	61.4	0.003
	Once a day	497	24.8	41	17.6	
	4–6 days /week	286	14.3	29	12.5	
	1–3 days /week	135	6.7	15	6.4	
	less than once a week	21	1.1	3	1.3	
	have not eaten yet	1	0.1	2	0.9	
fruit	≥ 2 times per day	208	10.4	34	14.6	0.306
	Once a day	555	27.7	60	25.8	
	4–6 days /week	543	27.1	66	28.3	
	1–3 days /week	531	26.5	51	21.9	
	less than once a week	162	8.1	21	9.0	
	have not eaten yet	5	0.3	1	0.4	
milk	≥ 2 times per day	706	35.2	103	44.2	0.002
	Once a day	718	35.8	91	39.1	
	4-6 days /week	305	15.2	20	8.6	
	1-3 days /week	203	10.1	18	7.7	
	less than once a week	52	2.6	1	0.4	
	have not eaten yet	20	1.0	0	0.0	
Processed food						
sweetened beverage	≥ 2 times per day	206	10.3	28	12.0	0.814
	Once a day	407	20.3	52	22.3	
	4–6 days /week	310	15.5	33	14.2	
	1–3 days /week	666	33.2	69	29.6	
	less than once a week	371	18.5	46	19.7	
	have not eaten yet	44	2.2	5	2.2	
confectionery	≥ 2 times per day	241	12.0	33	14.2	0.481
	Once a day	970	48.4	104	44.6	
	4-6 days /week	364	18.2	38	16.3	
	1-3 days /week	321	16.0	40	17.2	
	less than once a week	97	4.8	17	7.3	
	have not eaten yet	11	0.6	1	0.4	
instant noodle	≥ 2 times per day	0	0.0	0	0.0	0.082
	Once a day	4	0.2	1	0.4	
	4–6 days /week	13	0.7	2	0.9	
	1–3 days /week	187	9.3	23	9.9	
	less than once a week	1451	72.4	150	64.4	
	have not eaten yet	349	17.4	57	24.5	
fast food	≥ 2 times per day	0	0.0	0	0.0	0.014
	Once a day	5	0.3	1	0.4	
	4–6 days /week	15	0.8	3	1.3	
	1–3 days /week	232	11.6	17	7.3	
	less than once a week	1642	81.9	188	80.7	
	have not eaten yet	110	5.5	24	10.3	
		lsmean	SE	lsmean	SE	
Food diversity score†	8 points/day	3.64	0.19	4.04	0.22	0.002
Processed food score †	4 points/day	0.57	0.08	0.61	0.09	0.474

p: χ^2^ test

†: ANCOVA

†: adjusted relationship for the child, sex of child, employment status of mother, family living together, subjective economic status and leisure time, caregiver of the child during the day

Food diversity score: the total number of 8 food groups (grain, fish, meat, eggs, soybeans and soy products, vegetables, fruit and milk) eating at least once a day

Processed food score: the total number of 4 food items (sweetened beverage, confectionery, instant noodle and fast food) eating at least once a day

## Discussion

### Relationship between cooking meals together and parental concerns about the dietary habits of children

In the “National nutrition survey on preschool children” conducted in Japan [5], the parental concerns about the eating behaviors of children included “picky eating” and “playing with food/utensils while eating. Our study results confirmed that parents and children cooking meals together was associated with a lack of concern about children “picky eating” or “playing with food/utensils while eating,” but these parents were more likely to be concerned about children “eating too much” compared with parents who did not cook a meal together with their children. Thus, while in the conclusion we can speculate that cooking together may alleviate or increase the concerns of parents, this would have to be tested in interventional or experimental studies to explore it further.

The results of a previous systematic review highlighted the impact of parental involvement in dietary interventions to improve the dietary habits of children [12]. Family-based child nutrition programs are beneficial to all children irrespective of socioeconomic status [11, 12] and may be important to expand daily participation in meal preparation [11, 14, 15].

A previous study reported that “cooking programs for parents and children through home visits for low income families” [11] and “responsible for child’s cooking” could improve picky eating in a family intervention setting [13]. These findings were similar to those of the present study. However, relatively few studies included school-aged children [12]. Hence, further research is needed.

Some studies conducted in other countries reported that picky eating was related to food intake [20]. A study conducted in Japan in 1998 reported that the nutritional education approach involving children and parents cooking together improved the dietary habits of children [21]. Although later studies demonstrated increased motivation for food intake owing to the parent cooking together with school-aged children [22, 23], few studies addressed early childhood. Therefore, the results obtained from the analysis of our study are valuable.

An intervention study conducted in the United States reported that parent–child communication is benefited by preparing meals together because the children have the opportunity to ask their parents about the ingredients of healthy lunch recipes that are provided in school lunch programs [24]. Another study reported that liking of different foods of children was determined by food-related behaviors learned at a young age [25].

The health and development of children are influenced by conversations with parents during meal preparation [26, 27] and the child feeding practices of the parents [28], which may alleviate some parental concerns. In our study, the concerns of children were expressed from the perspective of the parents. For example, even if a parent cooks a balanced meal, the children sometimes may still refuse to eat. Consequently, the parents become concerned about the health of their children. In other words, the background of the parents’ concern is not only the health of their children but also their attitude towards eating [29], which may be a problem related to parent–child communication [30]. Future studies are warranted to deeply analyze the attitudes and behaviors of parents and children regarding the diet quality of children [31].

In addition, cooking together was related to leisure time, but not subjective economic status, employment status of mother, or the place where the children spend their daytime. In other words, it seems that there are other determinants for cooking a meal together. The attempts of a parent to cook meals with their children may be related to whether the parent enjoys cooking. In previous studies, it was reported that those who enjoyed cooking possessed the requisite skills [32] and were less likely to use processed or pre-cooked foods [33]. In addition, some studies reported the need for education and support of cooking skills based on the socioeconomic status of the household [34]. Enjoying cooking may be important to encourage parents to cook together with their children [33].

### Relationship between cooking a meal together and the food intake of children

The group of cooking together exhibited more frequent intake of fish, soy/soy products, vegetables, and milk, which were reported to have a low intake frequency in the “National nutrition survey on preschool children” in Japan [5].

Our study showed that parent–child cooking a meal together was related to adequate food intake, including tasting a diversity of foods. In a study by Allirot X et al., it was showed that involving children in cooking can increase their willingness to taste novel foods and direct food choices towards foods containing vegetables [35]. Previous studies reported that providing early education in the place where the children spent their days, such as nursery school, kindergarten, etc., was effective in decreasing the intake of an unbalanced diet [35, 36]. Our study identified that parent–child cooking a meal together at home could be important for healthy food intake by children.

In Health Japan 21 (second edition), the health of the next-generation incorporated indicators of eating behavior, such as “eating three meals a day” and “eating together,” but there was no indicator of behaviors related to meal preparation [36]. To encourage healthy development in early childhood, it is important to improve not only the eating behavior but also the ability to cook meals [10, 37]. Based on the results of our study, interventions to support parent–child cooking a meal together may be effective. Furthermore, it may also be vital to collaborate with the community and plan educational strategies to improve the cooking capabilities of both children and their parents [38, 39].

In the present study, parent–child cooking together was related to dietary habits of children and food intake, but the behavioral factors of parents related to the eating behaviors of children were not analyzed. Further studies on the relationship between parental behaviors, dietary concerns of the child, and food intake of the child are warranted.

There were several limitations to this study that should be addressed. First, the response rate was only 56.8%. Hence, we relied on the 2015 database of the National nutrition survey on preschool children conducted by the MHLW. In this investigation, 3871 questionnaires were collected from 3936 children. However, only 2237 participants responded to all of the required items. The most unanswered items were height, weight, and subjective economic status. Although height and weight are measurable, it may have been difficult for some parents to subjectively gauge their economic status. Since this survey is conducted every 10 years, it is necessary to devise a method to increase the response rate for such items in future surveys. Second, it is unknown whether the parental concerns about the dietary habits of children are related to childhood development. Furthermore, children may help with cooking because of the lack of motivation of the parents. Therefore, further research is required [6, 11]. Third, items related to the dietary habits of the parents were limited. Despite these limitations, parental concerns about the dietary habits of children may be related to cooking a meal together with young children.

## Conclusions

Cooking a meal together with a child may be related to the parent’s lower concerns about the dietary habits of the child, including “picky eating” and “playing with food/utensils while eating,” but may also be related to the higher concerns of “eating too much.”

## Additional file

Additional file 1: Study population and procedure diagram of this study. (PDF 150 kb)

## Data Availability

Permission for the use of the dataset in the current study was obtained from the MHLW, Japan. All data belong to the MHLW and the database cannot be used for other studies.

## Abbreviations

CI: Confidence interval
Lsmeans: Least squares mean
MCH: Maternal and child health
MHLW: Ministry of Health, Labour and Welfare
OR: Odds ratio
SD: Standard deviation
SE: Standard Error
