# Supplementary figures and images for: Parent–child cooking meal together may relate to parental concerns about the diets of their toddlers and preschoolers: a cross-sectional analysis in Japan
Source: Nutr J. 2019 Nov 18;18:76. doi: 10.1186/s12937-019-0480-0 (PMC6862729; doi:10.1186/s12937-019-0480-0)

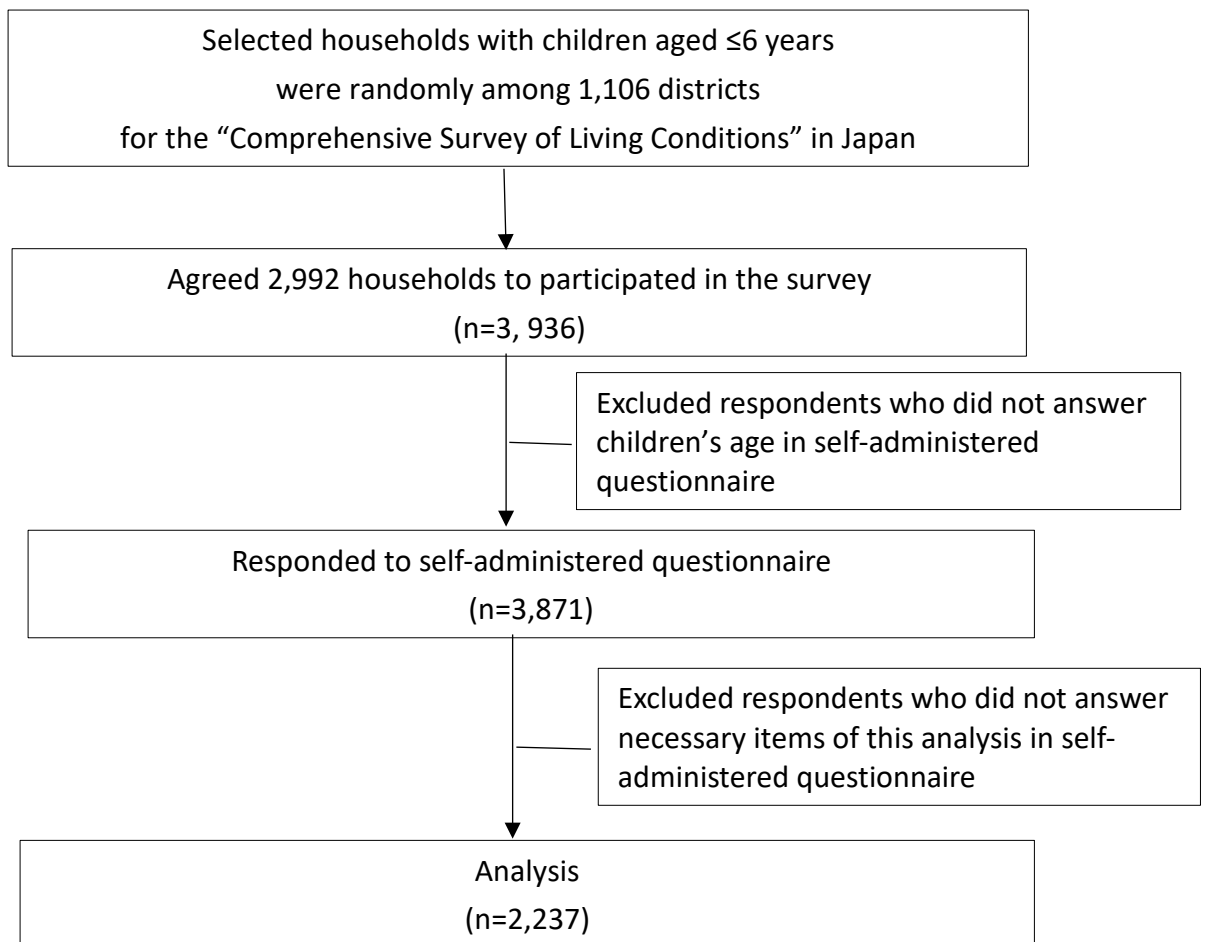

Study population and procedure diagram of this study

Supplement: Supplementary file 1 — Study population and procedure diagram of this study. (PDF 150 kb) [file 12937_2019_480_MOESM1_ESM.pdf]
